# Effects of a Guided Web-Based Smoking Cessation Program With Telephone Counseling: A Cluster Randomized Controlled Trial

**DOI:** 10.2196/jmir.3536

**Published:** 2014-09-24

**Authors:** Michael Mehring, Max Haag, Klaus Linde, Stefan Wagenpfeil, Antonius Schneider

**Affiliations:** ^1^Institute of General PracticeKlinikum rechts der IsarTechnische Universität MünchenMunichGermany; ^2^Institute for Medical Biometry, Epidemiology und Medical Informatics (IMBEI)Universitätsklinikum des Saarlandes, Homburg/SaarHomburgGermany

**Keywords:** smoking cessation, Web-based, randomized controlled trial, primary care

## Abstract

**Background:**

Preliminary findings suggest that Web-based interventions may be effective in achieving significant smoking cessation. To date, very few findings are available for primary care patients, and especially for the involvement of general practitioners.

**Objective:**

Our goal was to examine the short-term effectiveness of a fully automated Web-based coaching program in combination with accompanied telephone counseling in smoking cessation in a primary care setting.

**Methods:**

The study was an unblinded cluster-randomized trial with an observation period of 12 weeks. Individuals recruited by general practitioners randomized to the intervention group participated in a Web-based coaching program based on education, motivation, exercise guidance, daily short message service (SMS) reminding, weekly feedback through Internet, and active monitoring by general practitioners. All components of the program are fully automated. Participants in the control group received usual care and advice from their practitioner without the Web-based coaching program. The main outcome was the biochemically confirmed smoking status after 12 weeks.

**Results:**

We recruited 168 participants (86 intervention group, 82 control group) into the study. For 51 participants from the intervention group and 70 participants from the control group, follow-up data were available both at baseline and 12 weeks. Very few patients (9.8%, 5/51) from the intervention group and from the control group (8.6%, 6/70) successfully managed smoking cessation (OR 0.86, 95% CI 0.25-3.0; *P*=.816). Similar results were found within the intent-to-treat analysis: 5.8% (5/86) of the intervention group and 7.3% (6/82) of the control group (OR 1.28, 95% CI 0.38-4.36; *P*=.694). The number of smoked cigarettes per day decreased on average by 9.3 in the intervention group and by 6.6 in the control group (2.7 mean difference; 95% CI -5.33 to -0.58; *P*=.045). After adjustment for the baseline value, age, gender, and height, this significance decreases (mean difference 2.2; 95% CI -4.7 to 0.3; *P*=.080).

**Conclusions:**

This trial did not show that the tested Web-based intervention was effective for achieving smoking cessation compared to usual care. The limited statistical power and the high drop-out rate may have reduced the study’s ability to detect significant differences between the groups. Further randomized controlled trials are needed in larger populations and to investigate the long-term outcome.

**Trial Registration:**

German Register for Clinical Trials, registration number DRKS00003067; http://drks-neu.uniklinik-freiburg.de/drks_web/navigate.do?navigationId=trial.HTML&TRIAL_ ID=DRKS00003067 (Archived by WebCite at http://www.webcitation.org/6Sff1YZpx).

## Introduction

Tobacco smoking is a major preventable cause of death worldwide. The use of tobacco is estimated to kill 5.4 million people a year. By 2030, tobacco will contribute to the deaths of more than 8 million people a year. The overwhelming majority of those deaths are predicted to occur in the developing world [1]. Tobacco smokers are more prone to develop cancer and are also at substantially increased risk of developing heart disease, stroke, emphysema, and other severe diseases [2]. Prevention and cessation are the two principal strategies against tobacco smoking. Surveys are already indicating that almost 70% of smokers would like to stop smoking completely [3,4]. There is evidence that cessation advice given by a doctor is an efficient way to support smokers to quit and that more intensive interventions in general practice increases the abstinence rate [5]. Especially for younger smokers, the Internet may provide a vehicle to support this approach [6]. There were 2.4 billion Internet users worldwide with an increasing trend in 2012 [7]. The use of Web-based smoking cessation material provides low costs per user and results in high cost-effectiveness [8]. Web-based programs are convenient for users because the content can be accessed easily anytime and anywhere. For some people, the greater level of anonymity online than in in-person counseling may be appealing.

The evidence of a variety of computer and other electronic aids were summarized in a meta-analysis by Chen et al [9]. They concluded that computer and other electronic aids increase—to a small extent—the likelihood of prolonged smoking cessation compared with no intervention. A recently published Cochrane review [10] came to the conclusion that some Web-based interventions can assist smoking cessation, particularly those that are interactive and tailored to individuals. However, there were no consistent effects detected from trials that compared Internet interventions with usual care. Likewise, most of the included trials relied only on self-reported smoking status. Biochemical validation of self-reported cessation was only attempted in six of 28 trials.

Therefore, the aim of the present study was to compare the short-term effectiveness of a Web-based coaching program in combination with telephone counseling to usual care (see Multimedia Appendix 1). The Web-based program we tested, HausMed, combines an individually tailored strategy for smoking cessation with automated advice and feedback elements, in addition to monitoring via Internet and telephone counseling in general practice. Such a tool would facilitate the management of patients as they receive support from their general practitioner (GP) during the primary care process guided by the Web-based program. To date, very few findings within primary care patients and involving general practitioners are available. Additionally, the biochemical validation of the self-reported cessation status by a cotinine urine test was implemented in the present investigation to confirm the documented main outcome.

## Methods

### Design

The study was designed as a two-armed, unblinded cluster-randomized controlled trial. At the beginning of the study, around 2000 Bavarian general practitioners (GPs) received a fax by the Bavarian Association of General Practitioners with information about the research project. All interested GPs were sequentially registered for randomization. After giving written consent, the participating practices were randomized either to the intervention or the control arm. The sequence of randomization (allocation 1:1) was provided by a methodologist, who did not participate in the execution of the study, via the program Research Randomizer [11]. Randomization was concealed by using sequentially numbered, opaque, sealed envelopes held by the study coordinator. Before starting the recruitment of patients, physicians and practice nurses received detailed instructions by the research team on the study process (both intervention and control group) and on the coaching program (only intervention group).

Physicians assigned to the control arm were asked to change nothing in their usual way of counseling and to treat participants in the same manner as if they would have been non-participants. There was no structured documentation of the care provided. The patients recruited by intervention practices received free access to the Web-based coaching program. The patients of practices participating in the control arm were advised by the GPs in their individual way of usual care to quit smoking. The study was approved by the Medical Ethics Committee of the Technische Universität München (April 19, 2011) and was in accordance with ethical standards for human experimentation established by the Declaration of Helsinki. All participants gave written informed consent. A data and safety monitoring board was established before the beginning of the study.

### Participants and Procedures

Participating physicians were general practitioners in Bavaria, Germany. The GPs were requested to recruit individuals with the desire for smoking cessation (see Multimedia Appendix 2). Individuals at least 18 years old and with Internet access were potentially eligible. Exclusion criteria were aged younger than 18 years, insufficient German language skills, and lack of Internet access. Further exclusion criteria were psychiatric disorders and posttraumatic stress disorder.

After the GP decided that patient should participate, an information form was given and discussed with the patients, and a participation form had to be signed. At the same time, the baseline data acquisition took place. All participants were asked to fill in a standardized questionnaire together with the GP. The standardized questionnaire comprised the following information: age, sex, height, weight, physical activity, years of tobacco use, number of cigarettes smoked per day, number of previous quit attempts, use of current nicotine replacement therapy (NRT), and reasons for smoking. Participants of the intervention group received a free Web-code. The physician filled in a form together with the patient with information about the potential existence and grade of a chronic obstructive pulmonary disease. This form with the Internet code was used by the patient for specification during the registration process of the Internet program. Participants of both groups were requested after 12 weeks to document the follow-up evaluation together with their physician. The follow-up comprised again information about smoking status, weight, physical activity, number of cigarettes smoked per day, and use of current NRT. At follow-up, a biochemical validation of the self-reported cessation status was also implemented through a cotinine urine test. Cotinine is detected in the urine for 2-4 days after the use of tobacco.

Physicians in the intervention group received €50 per participant for time and effort. Physicians in the control group received €25. Participants in the intervention group received free access to the smoking cessation program, which usually costs €79. Participants in the control group received €10 as an incentive to come into practice for follow-up investigation after 12 weeks. No methodical changes were made during the entire study period.

### Intervention

Anamnestic and health data were documented in a structured registration form including information about the potential existence and grade of a chronic obstructive pulmonary disease from the GP. The patient received a copy of this form in order to use the health data for subscribing via Internet into the HausMed coaching program [12] at home. A specific program was installed to allow the participants to log in without charge. After completion of a pre-assessment, the program generated individual coaching based on the given information of the physicians (registration form), the physical characteristics, and the everyday behavior of the participants.

The coaching program is based on the generally accepted principles of cognitive behavioral therapy and combined psychoeducation and motivational techniques with behavioral-therapeutic elements [13]; for example, education, realistic goal-setting, and individual resources, and in particular, the behavioral change theory targeted to smoking cessation by using inexpensive Internet and mobile technologies in combination with existing health care resources of GPs. The content of the coaching program aimed at achieving a lasting change of behavioral patterns with the help of individualized education, motivation, exercise guidance, daily SMS text messaging (short message service, SMS) reminders, self-monitoring via Internet and, finally, through active monitoring and approximately three telephone calls during the 12 weeks by the GPs or their staff. The framework of the program is based on the idea by Buchkremer and Batra, of the Department of Psychiatry and Psychotherapy, University of Tübingen, Germany [14,15]. The development and implementation of the Web-based smoking cessation program was carried out by WeCARE GmbH, Göttingen, Germany. The coaching program was subdivided into 12 different constitutive modules. Each module was performed for one week and contains particular tasks (Textbox 1), which were supported by corresponding daily SMS reminders. The participant had to perform a specific task each day and received a daily SMS regarding that specific task.

**Figure 1:**
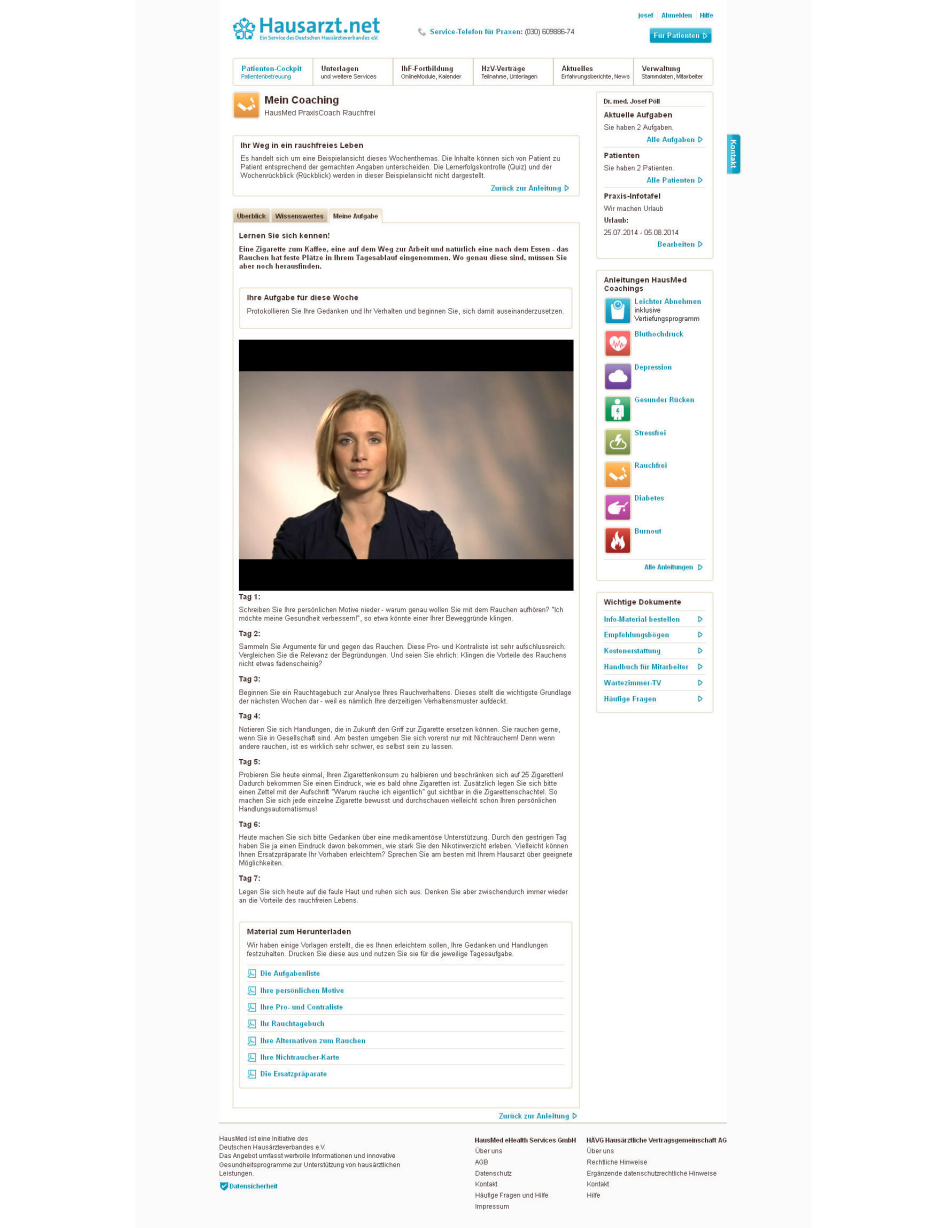
Specific daily tasks including interactive buttons, video clips, and learning progress quizzes.

**Figure 2:**
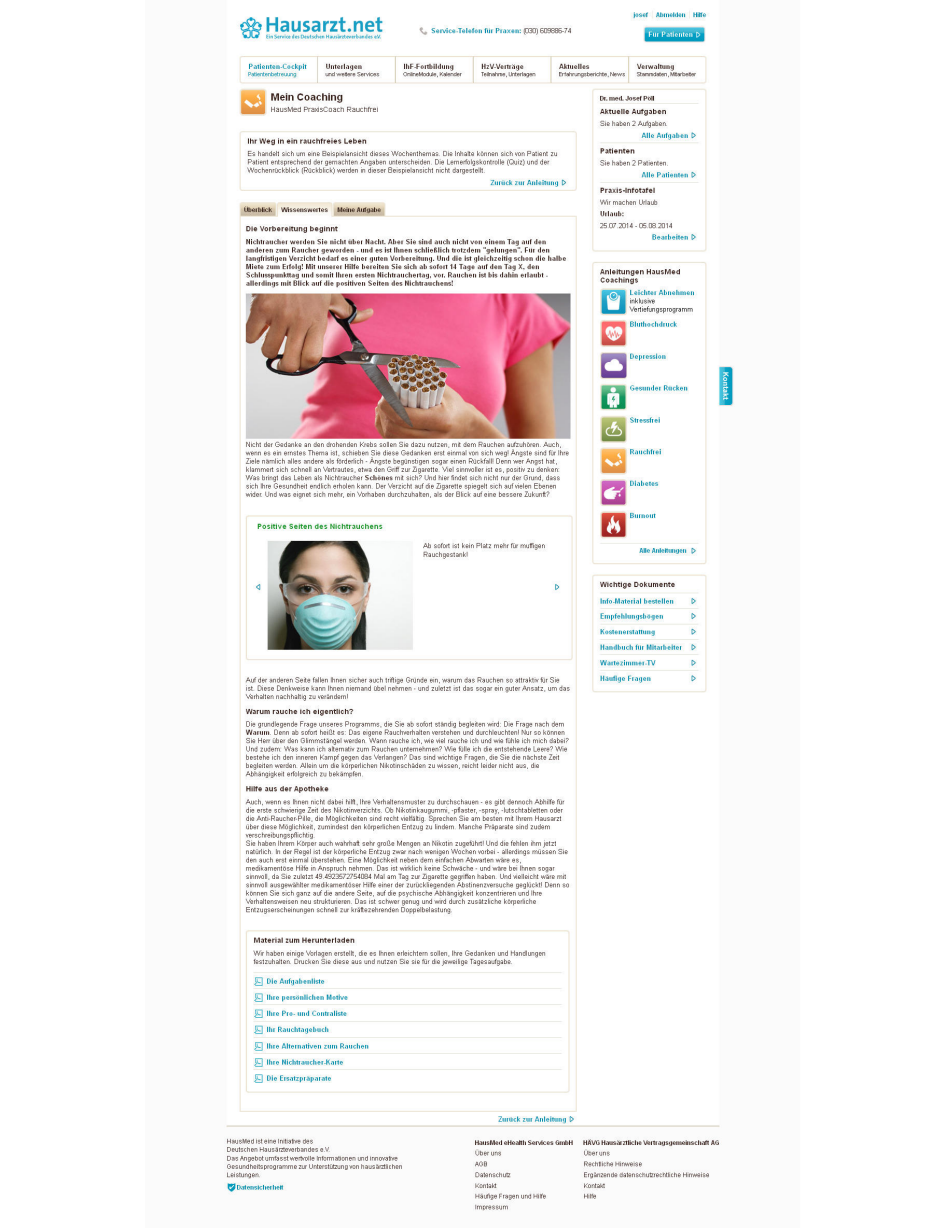
Sample printed materials: emergency plan, relaxation exercises, questionnaires, information, and self-agreements.

The reminder contained adapted information to maintain motivation, impart daily tips, and encourage daily performance of the respective task. The specific daily tasks were offered on the first day of each module. The coaching program also offered a variety of printed material (emergency plan, relaxation exercises, questionnaires, information, self-agreements, etc), which was connected to the respective task and included interactive buttons, video clips, and learning progress quizzes to examine learning success (Figures 1 and 2).

**Goals of the Web-based coaching program exercises.**

- Your path to a life without tobacco
- Get down to brass tacks
- This end is a beginning
- A look at the substitute bench
- To understand the withdrawal
- Reward pays twice
- Alternatively, for patients with COPD: Chronic bronchitis
- Food as an alternative?
- Relax without nicotine
- Race against addiction
- People are creatures of habit
- Alternative for light smokers: one is none
- With knowingness against the levity
- Alternative for stress-smoking: stress does not dissolve in smoke
- The journey is the destination

At the end of each week, participants were asked to give feedback via the Internet concerning their condition and level of motivation and whether or not they did their weekly tasks. Participants could also communicate among themselves on a forum or asked a HausMed team member in case they had any questions. The active monitoring (or rather supervising) of the entire 12-week coaching course was carried out by the GP through a separate login account in a secured physician area (motivation, condition, and status of the module exercise). In addition to that, three specified telephone calls from the GP or a qualified practice nurse (Weeks 2, 4, and 12) were implemented to motivate and support the participants. If either a participant’s motivation or condition declined notably at any point during the coaching period or if the module exercise was not completed, additional counseling from the GP or practice nurse was given over the telephone. There was no limitation to the frequency of website use, but participants were given a goal of using the website at least once a week and GPs were advised to log in into the program twice a week. No changes were made to the coaching program within the entire study period.

### Outcome Measures

The primary outcome measure was biochemically confirmed smoking status 12 weeks after inclusion into the study by use of a cotinine urine test. Secondary outcome measures were self-reported smoking status, number of NRTs, weight in kilograms, number of smoked cigarettes per day, physical activity (range from 0-4), and breathing difficulties (range from 0-4). A higher number on the scale refers to a more frequent physical activity and to more breathing difficulties. No changes were made on the trial outcome measures within the entire study period.

### Statistical Analysis

Sample size calculation was performed with G*Power 3 correcting for the cluster design (intracluster correlation coefficient=.05, average cluster size=3) for two-sided testing (alpha is 5% and a power of 80%). For the expected effect, abstinence rates of 30% versus 10% were assumed. Using these assumptions, the calculated total sample size for primary outcome smoking cessation was 152 participants. Taking expected attrition into account, we aimed at recruiting a total of 180 participants in about 80 general practices.

Baseline data are presented descriptively. Group differences were calculated for all participants whose smoking status was available at baseline and follow-up (completer collective). Sensitivity analysis was performed by an intent-to-treat analysis assuming that participants with missing values had no smoking status change at all. The strongly variable cluster size caused major numerical problems in the linear mixed model analysis. As it was not possible to adjust for intracluster correlations properly and because of the high variability of patients in practices, it was decided to perform the main analysis using Fisher’s exact test without accounting for the clusters. For the main outcome smoking status, we also performed secondary analyses based on logistic regression analyses with adjustments for age, gender, height, and number of cigarettes smoked per day. We further conducted generalized estimating equations as sensitivity analysis to account for practices as patient clusters. All analyses were performed using SPSS version 19.0.

## Results

**Figure 3:**
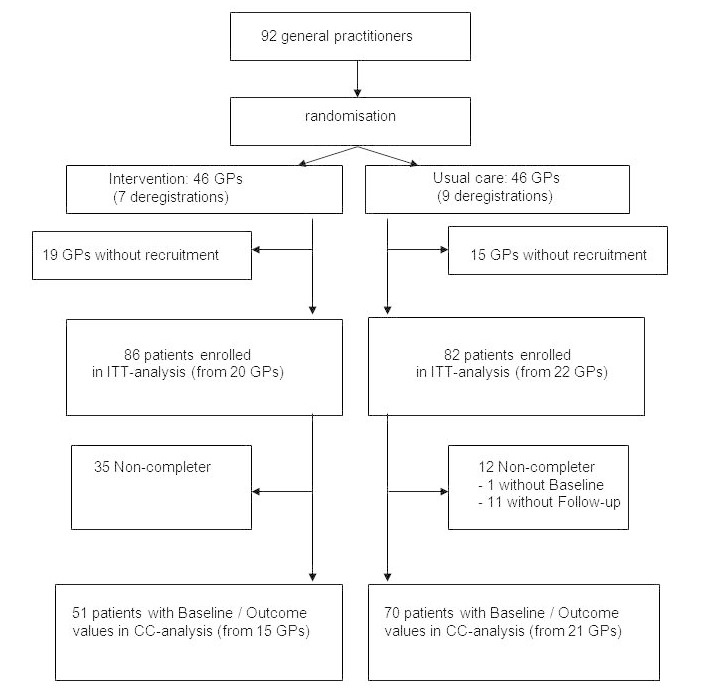
Participant flow of the study (GP=general practitioner; ITT=intent-to-treat; CC-Analysis=complete-case).

Originally 92 practices were interested in participating and were randomized. However, 16 practices withdrew early after randomization (7 GPs from the intervention and 9 GPs from the control group), and 34 practices (19 GPs from the intervention and 15 GPs from the control group) did not recruit any participants for the study (Figure 3). Altogether, 168 patients were recruited from 42 practices (86 patients in 20 intervention practices; 82 patients in 22 control practices) between May 19, 2011, and April 1, 2013. More than half of participants (54.5%, 66/121) were female, and the average age was 45.5 years. At 12 weeks, 35 participants in the intervention group did not show up for the measurement. In the usual care group, 12 participants had missing values at 12 weeks, and 1 of them had a missing baseline value. For 121 participants (51 from the intervention and 70 from the control group), information on smoking status was available both at baseline and after 12 weeks (complete-case). The proportion of non-completers (35/86; 12/82) was significantly higher (chi-square test, *P*<.001) in the intervention than in the usual care group.

The intervention and control group were similar in gender, age, weight, number of cigarettes smoked per day, and number of years with nicotine consumption. However, participants in the control group were significantly taller. A total of 7 participants used NRTs, and one participant in each group used varenicline at enrollment. There was no significant group difference found for the use of NRT or for the intake of varenicline (Table 1).

The self-reported cessation rate among the intervention group participants was 17.6% (9/51) and among the control group participants was 14.3% (10/70) without a significant group difference (*P*=.623). A logistic regression without adjustment (OR 0.78, 95% CI 0.29-2.08) and after adjustment for age, gender, and number of cigarettes smoked per day at baseline (OR 0.62, 95% CI 0.22-1.78) revealed similar results. Within the intent-to-treat analysis, self-reported cessation rate among the intervention group was 10.5% (9/86). The self-reported cessation rate of the control group was 11.3% (10/82). Results from the logistic regression without adjustment (OR 1.19, 95% CI 0.46-3.09) and after adjustment for age, gender, height, and number of cigarettes smoked per day at baseline (OR 1.04, 95% CI 0.39-2.80) were similar.

The results of the biochemical validation of the self-reported cessation status revealed that only a few (3/8) of the documented cotinine tests of the interventions group were positive, although they self-reported smoking cessation. None of these 3 participants were on nicotine replacement therapies, which would have explained the positive cotinine tests. The validation of the self-reported cessation status from the control group showed no disconfirmation. All six conducted cotinine tests were negative. No cotinine tests were administered for 1 participant of the intervention group and 4 participants of the control group who reported smoking cessation. The cessation rate by use of the biochemical validation was 9.8% for the intervention group (5/51) and 8.6% for the control group (6/70). Results were similar from the logistic regression without adjustment (OR 0.86, 95% CI 0.25-3.0) and after adjustment for age, gender, and number of cigarettes smoked per day at baseline (OR 0.63, 95% CI 0.17-2.40). The secondary analysis using a generalized estimating equation showed also a non-significant result (*P*=.74). Within the intent-to-treat analysis, the confirmed cessation rate among the intervention group was 5.8% (5/86) and for the control group 7.3% (6/82). Results from the logistic regression without adjustment (OR 1.28, 95% CI 0.38-4.36) and after adjustment for age, gender, and number of cigarettes smoked per day at baseline (OR 1.01, 95% CI 0.28-3.62) were similar (Table 2).

**Table 1:** Baseline characteristics at enrollment.

		Intervention			Usual care				
		n	mean	SD	n	mean	SD	Δ mean	*P*
Age, years		86	42.2	12.6	81	45.8	12.8	2.6	*.*072^a^
Weight, kg		85	73.3	15.7	81	78.0	17.2	4.3	*.*068^a^
Height, cm		85	169.8	8.7	81	173.5	9.3	4.2	*.*009^a^
Cigarettes smoked per day, n		86	18.2	7.4	81	16.6	9.4	2.8	*.*218^a^
Tobacco use, years		86	22.7	12.0	81	24.3	12.1	2.4	*.*410^a^
**Use of NRT**		4			3				1*.0* ^b^
	Nicotine patch	3			2				1*.0* ^b^
	Nicotine gum	1			1				1*.0* ^b^
Intake of varenicline		1			1				1*.0* ^b^
**Gender, n (%)**									*.*274^b^
	Females	51 (60.0)			40 (49.4)				
	Males	34 (40.0)			41 (50.6)				

^a^ *P* values from Student *t* test.

^b^ *P* values from Fisher’s exact.

**Table 2:** Results of self-reported and biochemically confirmed cessation status for intent-to-treat and complete-case analyses (*P* values are from Fisher’s exact test).

		Intent-to-treat					Complete-case				
		Intervention group		Usual care			Intervention group		Usual care		
		n	%	n	%	*P*	n	%	n	%	*P*
Self-reported cessation		9	10.5	10	12.2	.810	9	17.6	10	14.3	.623
**Not biochemically confirmed**											
	Relapse	77	89.5	72	87.8		42	82.4	60	85.7	
	Disconfirmed	3	3.5	0	0		3	5.9	0	0	
	Missing test	1	1.2	4	4.9		1	2	4	5.7	
Biochemically confirmed		5	5.8	6	7.3	.762	5	9.8	6	8.5	1.0
Total N		86	100	82	100		51	100	70	100	

The result from the secondary outcome, number of cigarettes smoked per day, revealed within the complete-case analysis that the mean difference after 12 weeks of the intervention group was 2.7 cigarettes less than the mean difference of the control group (95% CI -5.33 to -0.58; *P*=.045). After adjustment for number of cigarettes smoked per day at baseline, age, gender, and height, the difference between groups was no longer significant (95% CI 0.27-4.72; *P*=.080). There were no statistically significant group differences for other secondary outcomes like weight, physical activity, use of NRT, intake of varenicline, and breathing difficulties (Table 3). After 12 weeks, 8 participants used NRT and 4 participants used varenicline. One participant of the usual care group with a documented use of NRT did not further specify the used NRT. No participants using nicotine replacement had either a self-reported or biochemically confirmed cessation status.

Adverse events from 26 participants were documented. In the intervention group, 4 participants reported weight gain, 2 participants had increased perceived stress, 1 participant had a sleep disorder, and 1 participant had increased irritability. In the usual care group, 6 participants had increased perceived stress, 5 participants had cardiovascular problems, 4 participants reported fatigue, 4 participants reported weight gain, 2 participants had sweating, 1 participant had a sleep disorder, and 1 participant specified increased irritability. Additionally, one serious adverse event occurred that was not directly related to the intervention: a participant in the usual care group died due to a spontaneous rupture of an aortic aneurysm. This event was considered to be unrelated to the intervention and the study procedures.

**Table 3:** Results of NRT, breathing difficulties, physical activity, weight, and number of smoked cigarettes per day (complete-case analysis).

			Intervention			Usual care				
			n	mean	SD	n	mean	SD	Δ mean	*P*
**Use of NRT**										
	Baseline		4			3				.462^a^
		Nicotine patch	3			2				.651^a^
		Nicotine gum	1			1				1.0^a^
	After 12 weeks		4			4				.723^a^
		Nicotine patch	0			2				.511^a^
		Nicotine gum	4			1				.167^a^
**Intake of varenicline**										
	Baseline		1			1				1.0^a^
	After 12 weeks		1			3				.640^a^
**Breathing difficulties: (scale from 0 to 4)**										
	Baseline		51	1.0	1.2	70	1.0	1.0	0	.980^b^
	After 12 weeks		51	0.69	0.99	69	0.75	1.0	0.06	.704^b^
**Physical activity: (scale from 0 to 4)**										
	Baseline		51	1.4	1.0	70	1.4	1.0	0	.513^b^
	After 12 weeks		51	1.25	1.25	70	1.44	1.1	0.19	.231^b^
**Weight, kg**										
	Baseline		51	74.4	16.3	70	78.7	17.3	4.3	.169^b^
	After 12 weeks		48	74.8	17.2	67	80.1	17.2	5.3	.107^b^
**Number of smoked cigarettes per day**										
	Baseline		51	19.5	7.0	70	16.7	8.9	2.8	.063^b^
	After 12 weeks		51	10.2	7.7	69	10.2	8.3	-0.02	.989^b^
	Difference		51	-9.3	7.1	69	-6.6	7.3	2.7	.045^b^

^a^ *P* values from Fisher’s exact test.

^b^ *P* value from Student *t* test.

## Discussion

### Principal Findings

We found that the Web-based coaching program in combination with telephone counseling and monitoring in general practice was not effective for achieving smoking cessation compared to usual care.

Some trials have already shown that smoking cessation programs can be delivered effectively via the Internet [8,16-18], although Web-based interventions are often accompanied by a high attrition rate [19,20]. The evidence has been summarized in a recent meta-analysis by Chen et al [9]. They concluded that computer and other electronic aids increase to a small extent the likelihood of prolonged smoking cessation compared to no intervention. Another meta-analysis of Web- and computer-based smoking cessation programs indicates that there is currently sufficient evidence to support their use [21]. They stated within a meta-analysis of 22 randomized controlled trials (RCTs) that Web- or computer-based smoking cessation programs led to a 1.5 times higher abstinence rate compared to control groups. The effect of Web- or computer-based programs was therefore similar to that of counseling interventions. Their pooled cessation rate for Web- or computer-based programs over the long term (12 months) was 9.9% (95% CI 8.9-10.9). The advantage of a Web-based intervention compared to a control group cannot be confirmed by the present results, although our abstinence rates from both groups in the short term were comparable to theirs. In a Cochrane review, Civljak et al [10] also detected no consistent effects from trials that compared Internet interventions with usual care.

For example, Muñoz et al [22] suggested that quit rates obtained by using Internet interventions for smoking cessation are comparable with quit rates reported from smoking cessation therapies or smoking cessation groups. Our present result supports this fact as there was no significant difference between the intervention and the usual care in a primary care setting. Previous findings with a biochemical confirmation of the self-reported cessation support our results. Patten et al [23] revealed in adolescent smokers no significant treatment differences between a brief office intervention and a Web-based intervention. Their smoking abstinence rates after 24 weeks were 12% versus 6% for the brief office intervention and Internet-based intervention, respectively. The present discrepancy of the self-reported smoking status (17.6%) and the biochemical validation (9.8%) was noticeable even if this conspicuousness is based on only three individuals in the treatment group with a disconfirmed smoking status. It has been reported that biochemical validation does not modify the conclusions of low-intensity interventions trials [24]. Glasgow et al observed a disconfirmation rate between 4-5% over all groups. Compared to the present disconfirmation, we identified a similar rate only in the intervention group of 3.5-5.9%. In the control group, all conducted cotinine tests were negative so there was no disconfirmation to be documented. The self-reported cessation rate from 17.6% (complete-case) of the intervention group compared to the confirmed rate from 9.8%, beside the missing validation of 2% (n=1), leads to the suggestion of a certain overestimation from the present self-reported cessation rate. Nevertheless, this discrepancy could have still occurred by chance due to the small number of disconfirmed results.

The present study underlines how difficult it is to achieve smoking cessation in primary care and how difficult it is to motivate patients to quit smoking. Based on a parallel trial conducted with comparable conditions, we identified that a Web-based intervention with telephone counseling led to a significantly greater weight reduction [25] compared to usual care. This advantage was not shown regarding smoking cessation. Many reasons have been already identified to explain the low effectiveness of smoking cessation interventions in general practice. Patients’ low motivation to quit [26,27], patients’ low compliance [26,28], patients’ resistance to speak about smoking [29,30], lack of time [31,32], lack of economical reimbursement [28,33], lack of skills and low self-efficacy [32,34], consideration of smokers’ other problems [30,35], and an uneasy feeling when talking about smoking cessation [31,32] have been already identified as barriers. Notwithstanding, even interventions with a small effect are capable of substantially decreasing diseases that are associated with nicotine use [36].

### Strengths and Limitations

Strengths of the present study were the embedding of the study in a realistic primary care setting and the use of a biochemical validation of the self-reported cessation status. However, some important methodological aspects for the interpretation of the study results need to be considered. First, the randomization of the present study was conducted at the practice level before individual participants were included. Thus, physicians knew whether they recruited patients for the intervention or the control group, which could lead to bias. Second, due to the highly variable cluster sizes the statistical analysis of our data was not straightforward. Classical linear mixed models taking the cluster design into account could not be used because of numerical problems. Therefore, we used simple Fishers’ exact test (which ignores intracluster correlation) and an additional multilevel analysis (which runs into problems when cluster sizes differ) as sensitivity analysis. Third, according to our power calculations the target number of participants was not completely reached due to a slow recruitment of participants, and at a certain point the study had to be stopped, which may have reduced the study’s ability to detect significant differences between the groups. Fourth, the proportion of participants without follow-up values was undoubtedly higher in the intervention than in the usual care group. This could be partly due to the fact that participants in the control group received a small financial incentive while those in the intervention group did not. Participants in the intervention group might also have been less willing to have an additional practice visit after completing the program than those in the control group who had little practice contact otherwise. Therefore, our complete-case analysis with the self-reported cessation might overestimate the rates to some extent. Within the intent-to-treat analysis, where the missing post values were replaced without a change of smoking status (baseline carried forward), the cessation rates were clearly smaller. Fifth, the content of the usual care was not further evaluated. The practitioners of the control group were asked to change nothing in their usual way of counseling and to treat their participants in the same manner as usual. There was no additional documentation of their counseling provided.

### Conclusions

Our findings suggest that the tested Web-based coaching program in combination with telephone counseling and monitoring in general practice was not effective for achieving smoking cessation compared to usual care. The effect was similar to usual primary care and comparable to other Web-based interventions. We identified a discrepancy of self-reported smoking cessation and the biochemical validation, which should be reconsidered for further studies. The limited statistical power and the high drop-out rate may have reduced the study’s ability to detect significant differences between the groups. Further RCTs are needed in order to investigate long-term outcomes and interventions in larger populations, as well as in the contents of usual primary care.

## Multimedia Appendix 1

HausMed - Presentation Video.

## Multimedia Appendix 2

Waiting room advertisement.

## Multimedia Appendix 3

CONSORT-EHEALTH checklist V1 6.2 [37].

## Abbreviations

COPD: chronic obstructive pulmonary disease
GP: general practitioner
NRT: nicotine replacement therapy
RCT: randomized controlled trial
