# Supplementary material for: Effects of a Guided Web-Based Smoking Cessation Program With Telephone Counseling: A Cluster Randomized Controlled Trial
Source: J Med Internet Res. 2014 Sep 24;16(9):e218. doi: 10.2196/jmir.3536 (PMC4211026; doi:10.2196/jmir.3536)
Supplement: Supplementary file 2 [file jmir_v16i9e218_app2.ppt]

## Slide 1
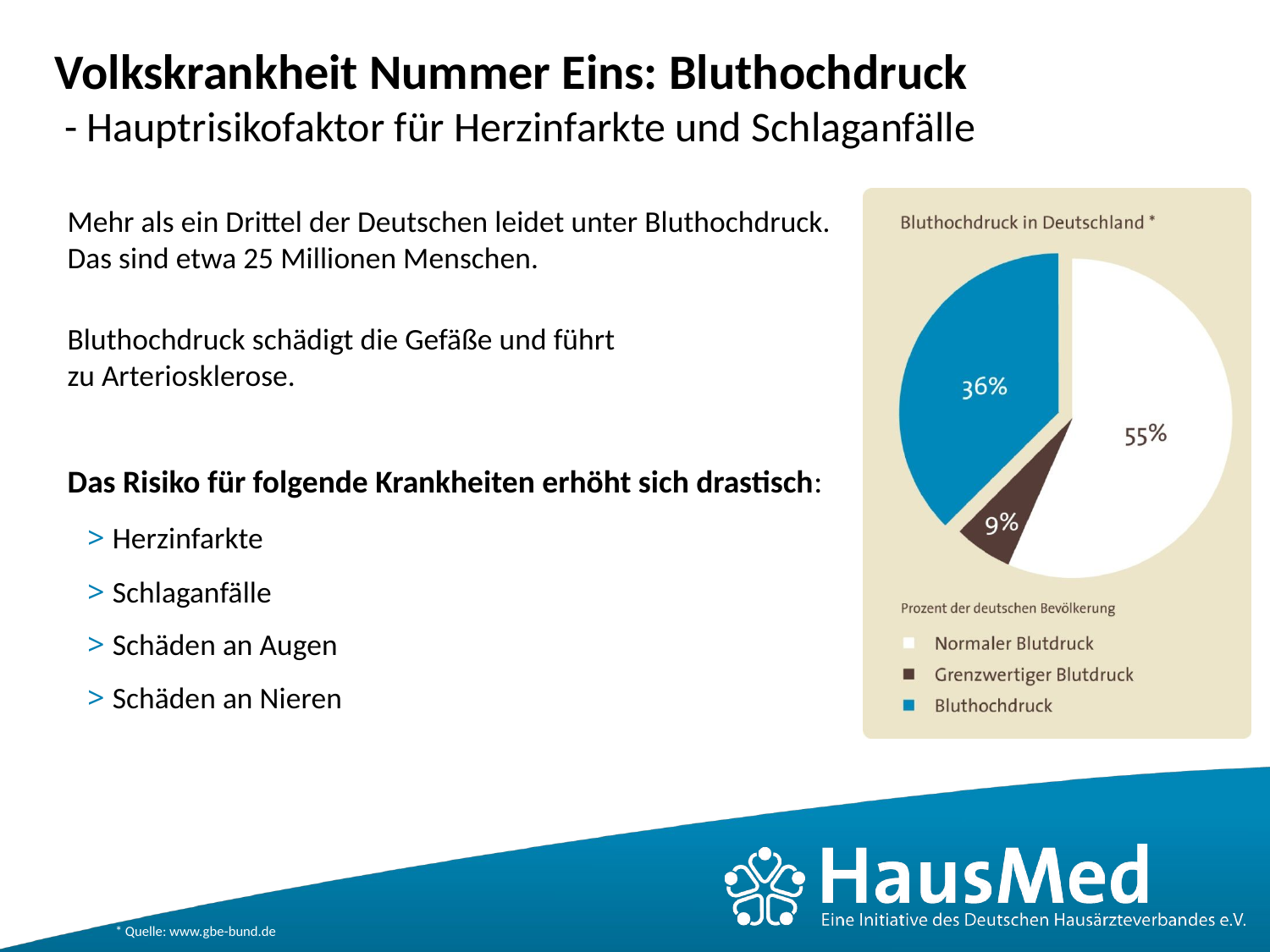

Volkskrankheit Nummer Eins: Bluthochdruck
 - Hauptrisikofaktor für Herzinfarkte und Schlaganfälle
Mehr als ein Drittel der Deutschen leidet unter Bluthochdruck.
Das sind etwa 25 Millionen Menschen.
Bluthochdruck schädigt die Gefäße und führt
zu Arteriosklerose.
Das Risiko für folgende Krankheiten erhöht sich drastisch:
> Herzinfarkte
> Schlaganfälle
> Schäden an Augen
> Schäden an Nieren
* Quelle: www.gbe-bund.de

## Slide 2
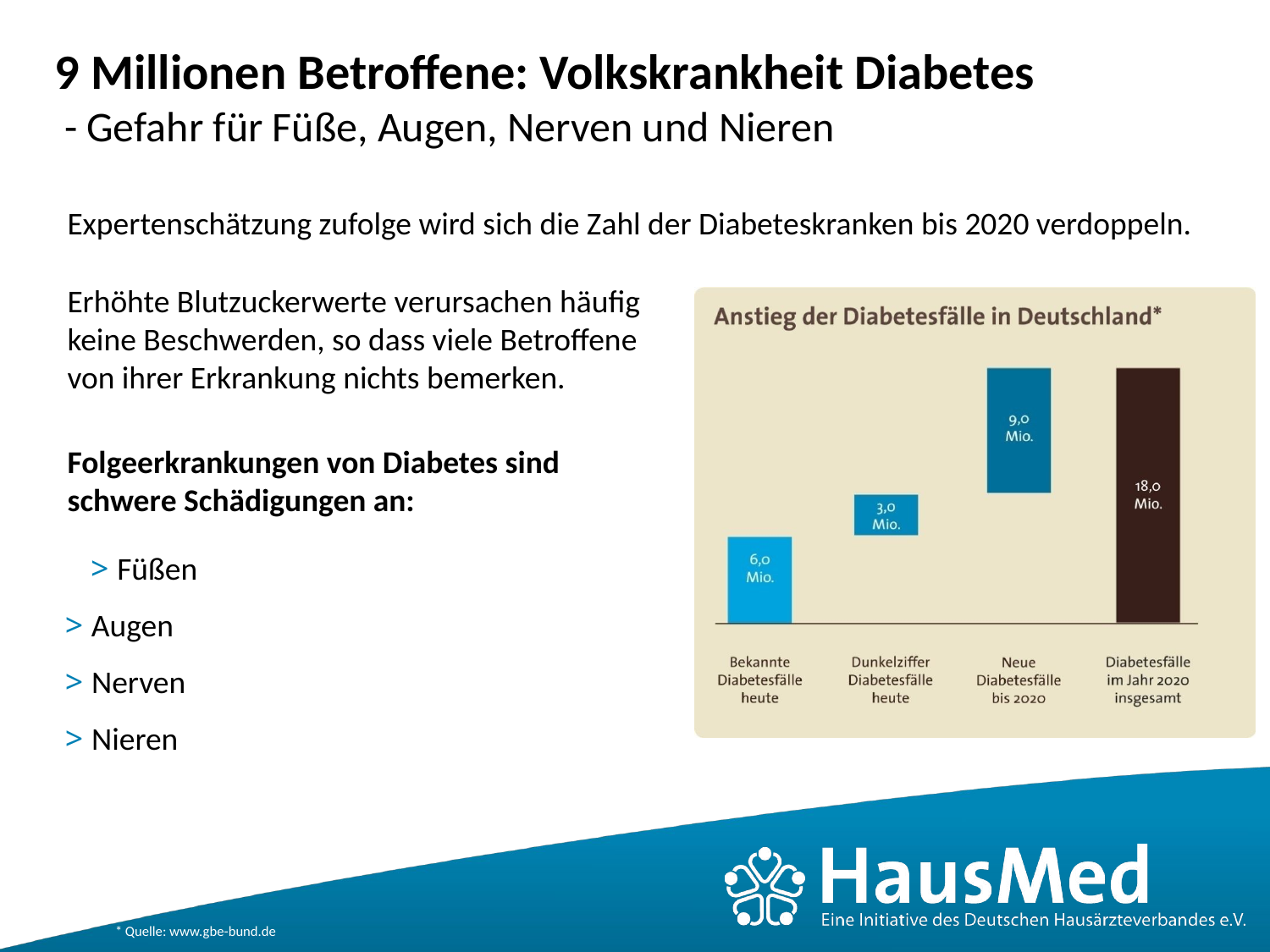

9 Millionen Betroffene: Volkskrankheit Diabetes
 - Gefahr für Füße, Augen, Nerven und Nieren
Expertenschätzung zufolge wird sich die Zahl der Diabeteskranken bis 2020 verdoppeln.
Erhöhte Blutzuckerwerte verursachen häufig
keine Beschwerden, so dass viele Betroffene
von ihrer Erkrankung nichts bemerken.
Folgeerkrankungen von Diabetes sind
schwere Schädigungen an:
> Füßen
> Augen
> Nerven
> Nieren
* Quelle: www.gbe-bund.de

## Slide 3
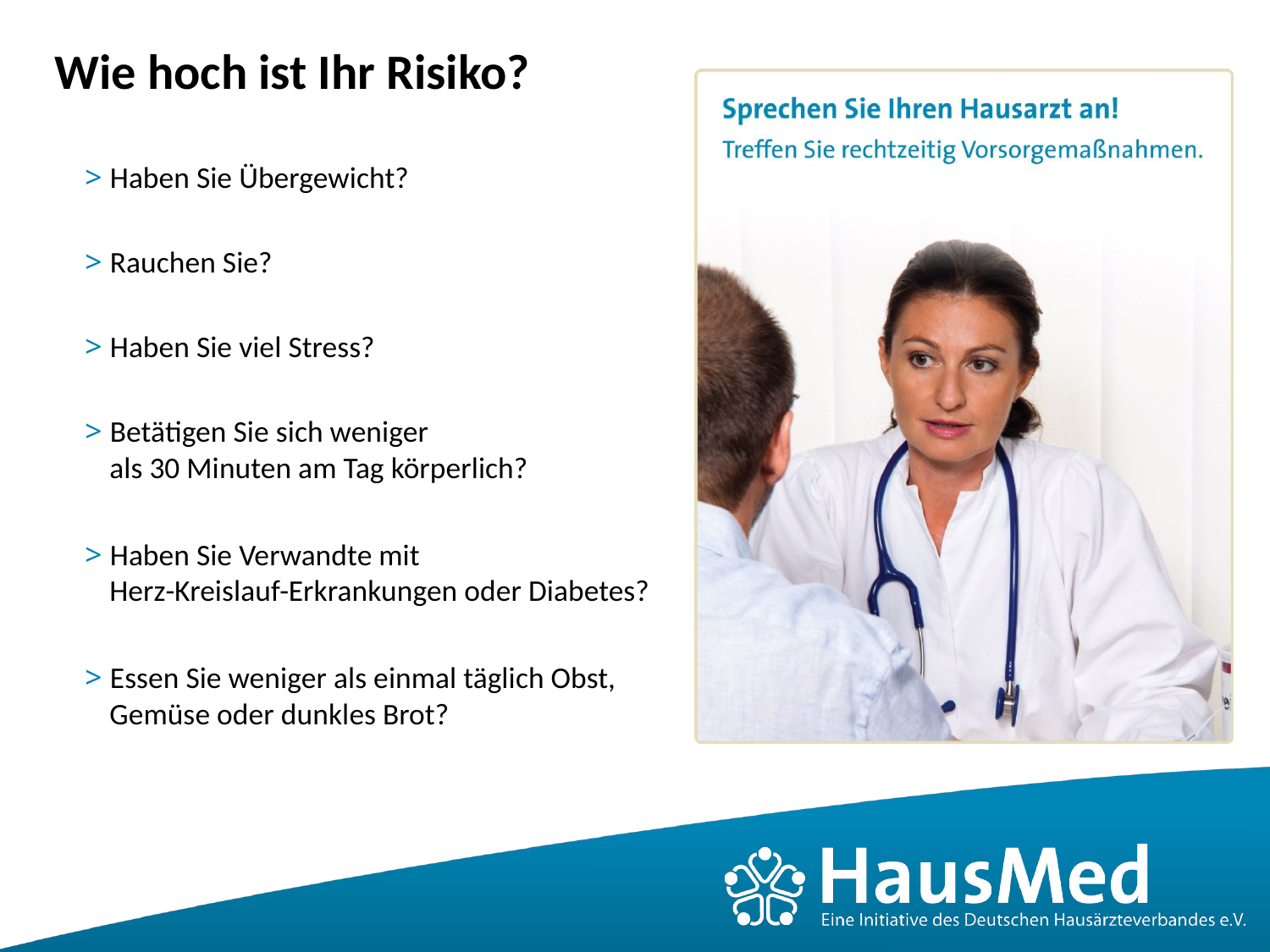

Wie hoch ist Ihr Risiko?
> Haben Sie Übergewicht?
> Rauchen Sie?
> Haben Sie viel Stress?
> Betätigen Sie sich weniger
als 30 Minuten am Tag körperlich?
> Haben Sie Verwandte mit
Herz-Kreislauf-Erkrankungen oder Diabetes?
> Essen Sie weniger als einmal täglich Obst,
Gemüse oder dunkles Brot?

## Slide 4
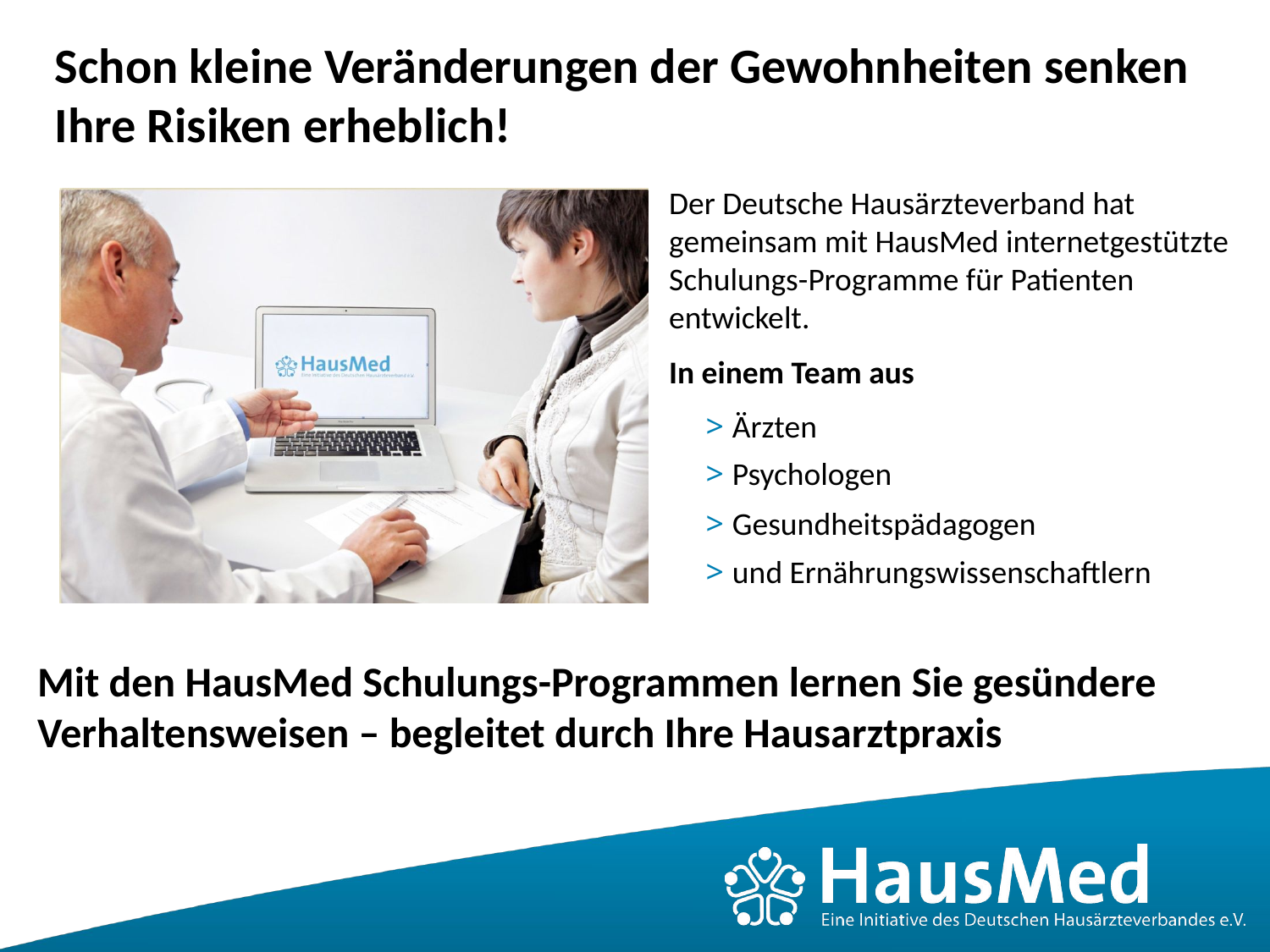

Schon kleine Veränderungen der Gewohnheiten senken Ihre Risiken erheblich!
Der Deutsche Hausärzteverband hat gemeinsam mit HausMed internetgestützte Schulungs-Programme für Patienten entwickelt.
In einem Team aus
> Ärzten
> Psychologen
> Gesundheitspädagogen
> und Ernährungswissenschaftlern
Mit den HausMed Schulungs-Programmen lernen Sie gesündere Verhaltensweisen – begleitet durch Ihre Hausarztpraxis

## Slide 5
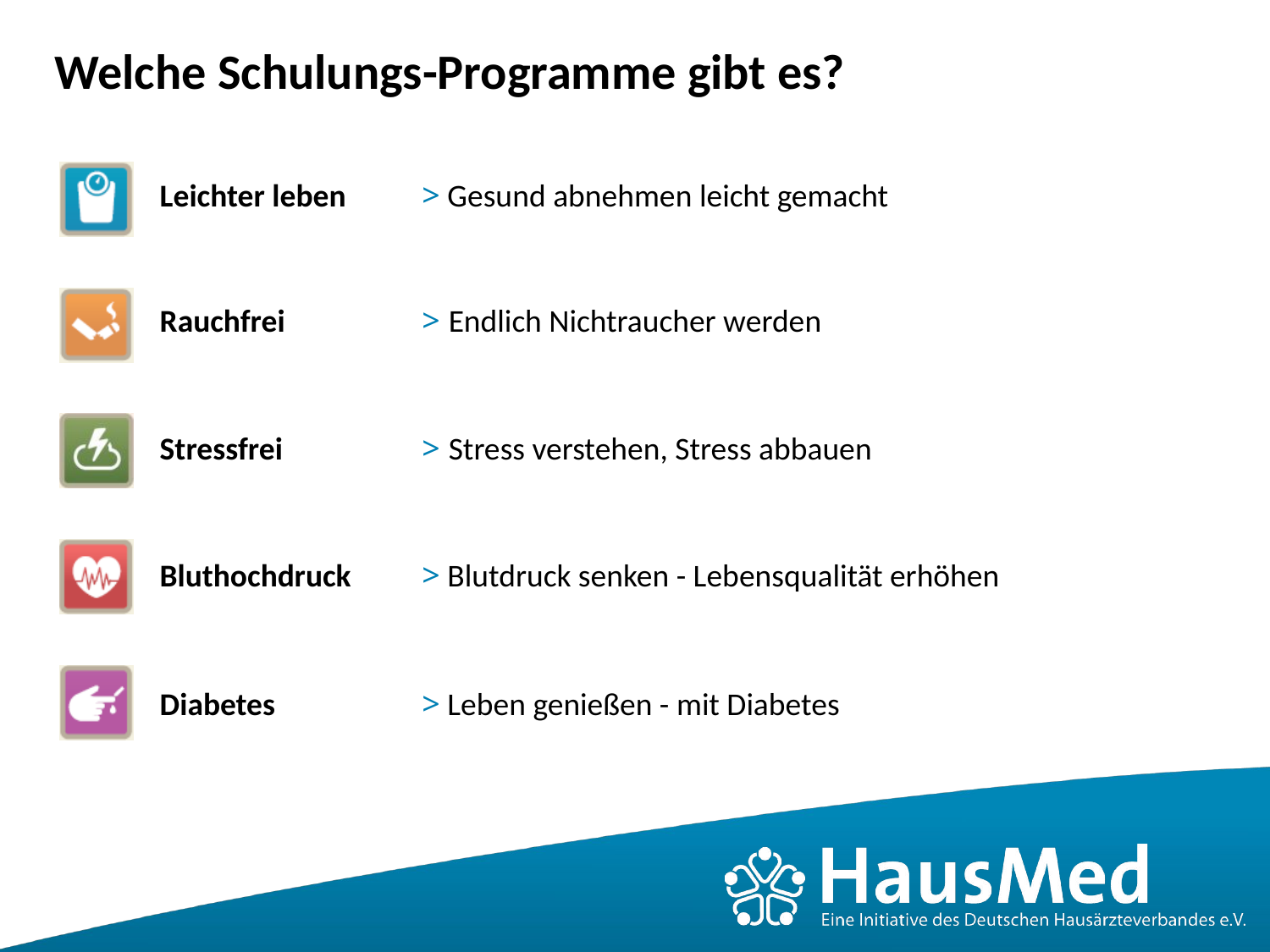

Welche Schulungs-Programme gibt es?
Leichter leben 		 > Gesund abnehmen leicht gemacht
Rauchfrei		 > Endlich Nichtraucher werden
Stressfrei 		 > Stress verstehen, Stress abbauen
Bluthochdruck 		 > Blutdruck senken - Lebensqualität erhöhen
Diabetes		 > Leben genießen - mit Diabetes

## Slide 6
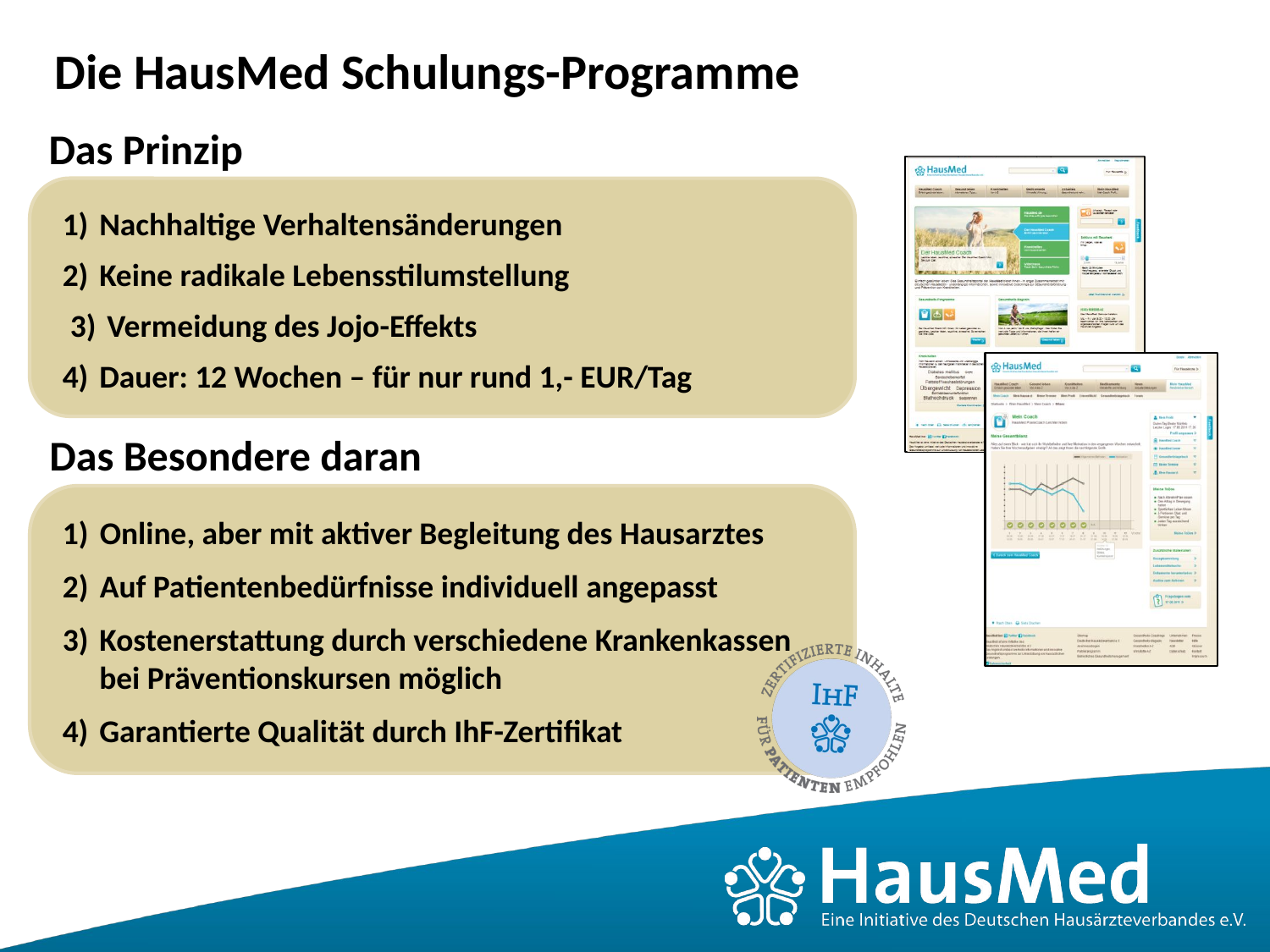

Die HausMed Schulungs-Programme
Das Prinzip
Nachhaltige Verhaltensänderungen
Keine radikale Lebensstilumstellung
Vermeidung des Jojo-Effekts
Dauer: 12 Wochen – für nur rund 1,- EUR/Tag
Das Besondere daran
Online, aber mit aktiver Begleitung des Hausarztes
Auf Patientenbedürfnisse individuell angepasst
Kostenerstattung durch verschiedene Krankenkassen bei Präventionskursen möglich
Garantierte Qualität durch IhF-Zertifikat

## Slide 7
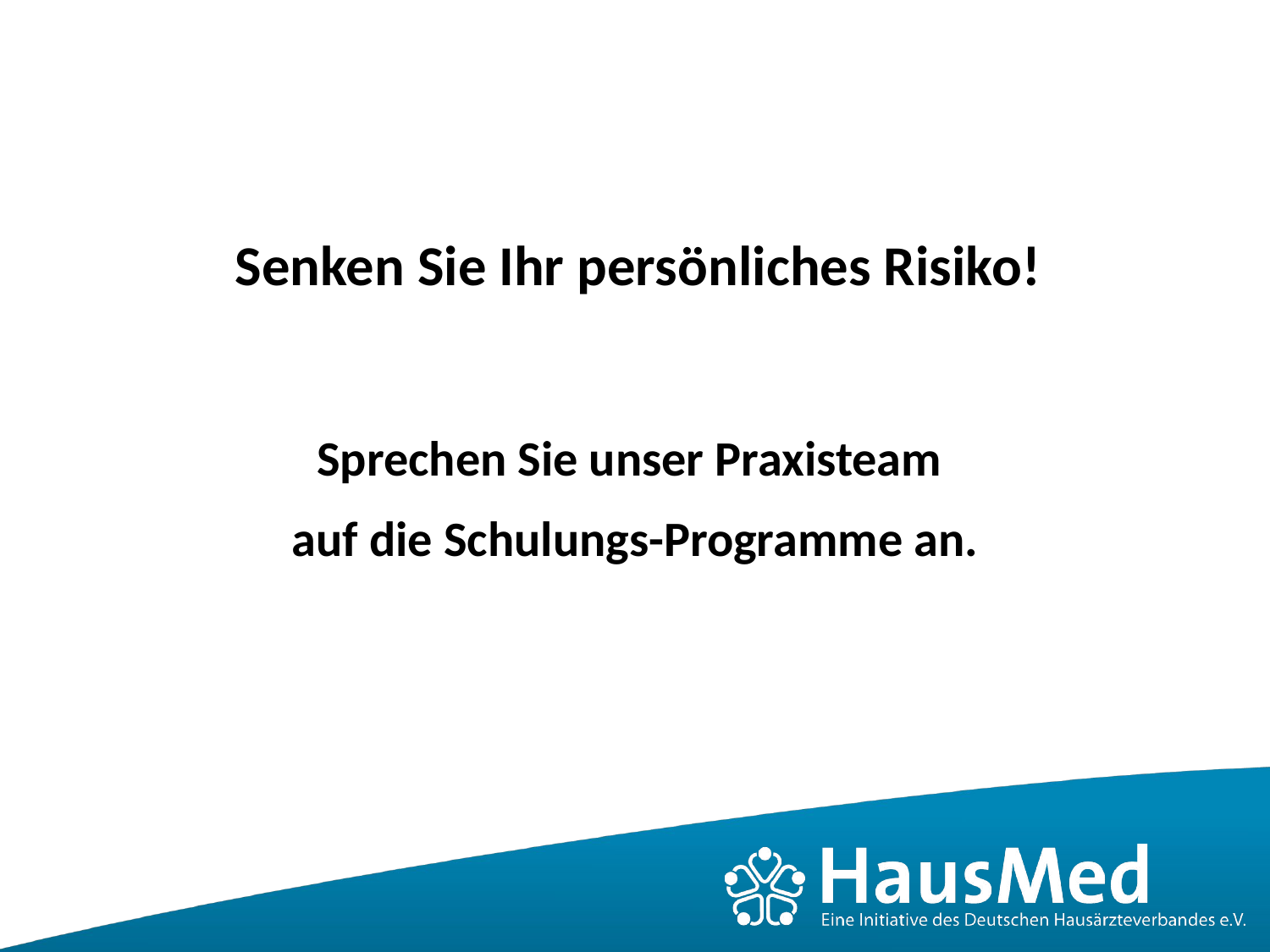

Senken Sie Ihr persönliches Risiko!
Sprechen Sie unser Praxisteam
auf die Schulungs-Programme an.
