# Supplementary material for: Effects of a Guided Web-Based Smoking Cessation Program With Telephone Counseling: A Cluster Randomized Controlled Trial
Source: J Med Internet Res. 2014 Sep 24;16(9):e218. doi: 10.2196/jmir.3536 (PMC4211026; doi:10.2196/jmir.3536)
Supplement: Supplementary file 3 [file jmir_v16i9e218_app3.pdf]

CONSORT-EHEALTH Checklist V1.6.2 Report

(based on CONSORT-EHEALTH V1.6), available at [http://tinyurl.com/consort-ehealth-v1-6].

3536

Date completed

5/12/2014 17:16:02

by

Michael Mehring

"Effects of a general practice guided web-based smoking cessation program with an accompanied telephone counselling - results of a cluster-randomized controlled trial"

TITLE

1a-i) Identify the mode of delivery in the title

"web-based smoking cessation program with an accompanied telephone counselling"

1a-ii) Non-web-based components or important co-interventions in title

"general practice guided web-based smoking cessation program with an accompanied telephone counselling"

1a-iii) Primary condition or target group in the title

"general practice guided"

ABSTRACT

1b-i) Key features/functionalities/components of the intervention and comparator in the METHODS section of the ABSTRACT

"... web-based coaching program based on education, motivation, exercise guidance, daily SMS reminding, weekly feedback through internet and active monitoring by general practitioners...Participants in the control group received usual care and advice from their practitioner without the web-based coaching program."

1b-ii) Level of human involvement in the METHODS section of the ABSTRACT

"All components of the program are fully automated."

1b-iii) Open vs. closed, web-based (self-assessment) vs. face-to-face assessments in the METHODS section of the ABSTRACT

"Individuals recruited by general practitioners randomized... Participants in the control group received usual care and advice from their practitioner without the web-based coaching program. Main outcome was the biochemically confirmed smoking status after 12 weeks."

1b-iv) RESULTS section in abstract must contain use data

"168 participants (86 intervention group, 82 control group) were recruited into study."

1b-v) CONCLUSIONS/DISCUSSION in abstract for negative trials

"This trial could not show that the tested web-based intervention was effective for achieving smoking cessation compared to usual care. The poor attendance and the missed power may have reduced the study’s ability to detect significant differences between the groups."

INTRODUCTION

2a-i) Problem and the type of system/solution

"However there were no consistent effects detected from trials that compared internet interventions with usual care. Likewise most of the included trials relied only on self-reported smoking status. Biochemical validation of self-reported cessation was only attempted in six from twenty-eight trials. Therefore, the aim of the present study was to compare the short-term effectiveness of a web-based coaching program in combination with an accompanied telephone counselling to usual care. The tested web-based program combines an individually tailored strategy for smoking cessation with automated advice and feedback elements, in addition to monitoring via internet and telephone counselling in general practice. Such tool would facilitate the management of patients as they receive support from their general practitioner (GP) during the primary care process guided by the web-based program. To date only few findings within primary care patients and especially the involvement of general practitioners are available. Additionally the biochemical validation of the self-reported cessation status by cotinine urine test was implemented in the present investigation to confirm the documented main outcome."

2a-ii) Scientific background, rationale: What is known about the (type of) system

"The evidence of a variety of computer and other electronic aids were summarized in a meta-analysis by Chen at al. [8]. They concluded that computer and other electronic aids increase to a small extent the likelihood of prolonged smoking cessation compared with no intervention. A recently published Cochrane review [9] came to the conclusion that some web-based interventions can assist smoking cessation, particularly those which are interactive and tailored to individuals. However there were no consistent effects detected from trials that compared internet interventions with usual care."

METHODS

3a) CONSORT: Description of trial design (such as parallel, factorial) including allocation ratio

"... the aim of the present study was to compare the short-term effectiveness of a web-based coaching program in combination with an accompanied telephone counselling to usual care...Additionally the biochemical validation of the self-reported cessation status by cotinine urine test was implemented in the present investigation to confirm the documented main outcome."

3b) CONSORT: Important changes to methods after trial commencement (such as eligibility criteria), with reasons

"No methodical changes were made during the entire study period."

3b-i) Bug fixes, Downtimes, Content Changes

"No changes were made on the coaching program within the entire study period."

4a) CONSORT: Eligibility criteria for participants

"Participating physicians were general practitioners in Bavaria, Germany. The GPs were requested to recruit individuals with the desire for smoking cessation and for whom smoking cessation was recommendable. Individuals with at least 18 years and internet access were potentially eligible. Exclusion criteria were age younger than 18 year, insufficient German language skills and lack of internet access. Further exclusion criteria were psychiatric disorders and posttraumatic stress disorder."

4a-i) Computer / Internet literacy

"Exclusion criteria were age younger than 18 year, insufficient German language skills and lack of internet access."

4a-ii) Open vs. closed, web-based vs. face-to-face assessments:

"The patients recruited by intervention practices received free access to the web-based coaching program. The patients of practices participating in the control arm were advised by the GPs in their individual way of usual care to quit smoking...The GPs were requested to recruit individuals with the desire for smoking cessation and for whom smoking cessation was recommendable...After decision of the GP that the participation of a patient was recommendable, an information form was given and discussed with the patients and a participation form had to be signed. At the same time the baseline data acquisition took place. All participants were asked to fill in a standardized questionnaire together with the GP. "

4a-iii) Information giving during recruitment

"Before starting recruitment of patients, physicians and practice nurses received detailed instructions by the research team on the study process (both intervention and control group) and on the coaching program (only intervention group)... After decision of the GP that the participation of a patient was recommendable, an information form was given and discussed with the patients and a participation form had to be signed...

4b) CONSORT: Settings and locations where the data were collected
"After decision of the GP that the participation of a patient was recommendable, an information form was given and discussed with the patients and a participation form had to be signed. At the same time the baseline data acquisition took place. All participants were asked to fill in a standardized questionnaire together with the GP. The standardized questionnaire comprised following information: age, sex, high, weight, physical activity, years of tobacco use, number of cigarettes smoked per day, number of previous quit attempts, use of current nicotine replacement therapy, reasons for smoking... Participants of both groups were requested to document after twelve weeks the follow-up evaluation together with their physician. The follow-up comprised again information about the smoking status, weight, physical activity, number of cigarettes smoked per day and use of current nicotine replacement therapy. At follow-up a biochemical validation of the self-reported cessation status was additional implemented by the use of a cotinine urine test."
4b-i) Report if outcomes were (self-)assessed through online questionnaires
"Participants of both groups were requested to document after twelve weeks the follow-up evaluation together with their physician... follow-up a biochemical validation of the self-reported cessation status was additional implemented by the use of a cotinine urine test."
4b-ii) Report how institutional affiliations are displayed
"At the beginning of the study, around 2.000 Bavarian general practitioners (GPs) received a fax by the Bavarian Association of General Practitioners with information about the research project...All participants gave written informed consent...The physician filled in a form together with the patient "
All used materials (internet and paper) displayed the affiliations from "Hausmed" and the "Technische Universtität München, Munich, Germany"
5) CONSORT: Describe the interventions for each group with sufficient details to allow replication, including how and when they were actually administered
5-i) Mention names, credential, affiliations of the developers, sponsors, and owners
"The patient received a copy of this form in order to use the health data for inscribing via internet into the coaching program (HausMed eHealth Services GmbH, Berlin; Germany; www.hausmed.de; indexed in internet archive) at home."
5-ii) Describe the history/development process
"The coaching program is based on principles of cognitive behavioural therapy – e.g. education, realistic goal-setting and individual resources – and in particular on the behavioural change theory targeted to smoking cessation by using inexpensive internet and mobile technologies in combination with existing health care resources of GPs."
For us is no further history or development process available.
5-iii) Revisions and updating
"No changes were made on the coaching program within the entire study period."
5-iv) Quality assurance methods
"The primary outcome measure was biochemically confirmed smoking status twelve weeks after inclusion into the study by use of a cotinine urine test."
5-v) Ensure replicability by publishing the source code, and/or providing screenshots/screen-capture video, and/or providing flowcharts of the algorithms used
"...HausMed eHealth Services GmbH, Berlin; Germany; www.hausmed.de; indexed in Internet Archive.." flowchart, screenshots and multimedia-appendix will be provide
5-vi) Digital preservation
"...HausMed eHealth Services GmbH, Berlin; Germany; www.hausmed.de; indexed in Internet Archive.."
5-vii) Access
"The physician filled in a form together with the patient with information about the potential existence and grade of a chronic obstructive pulmonary disease. This form with the internet-code was used by the patient for specification during the registration process of the internet program... Anamnestic and health data were documented in a structured registration form including information about a potential existence and grade of a chronic obstructive pulmonary disease from the GP. The patient received a copy of this form in order to use the health data for inscribing via internet into the coaching program ... at home. A specific website was installed for the participants to allow a login without charge."
We will provide a "backdoor" login account or demo mode.
5-viii) Mode of delivery, features/functionalities/components of the intervention and comparator, and the theoretical framework
"After completion of a pre-assessment, the program generated an individual coaching based on the given information of the physicians (registration form), the physical characteristics and the everyday behaviour of the participants...The coaching program is based on principles of cognitive behavioural therapy – e.g. education, realistic goal-setting and individual resources – and in particular on the behavioural change theory targeted to smoking cessation by using inexpensive internet and mobile technologies in combination with existing health care resources of GPs. The content of the coaching program aims at achieving a lasting change of behaviour patterns with the help of individualized education, motivation, exercise guidance, daily SMS reminders, self-monitoring via internet and, finally, through an active monitoring and approximately 3 telephone calls during the 12 weeks by the GPs or their respective staff. The coaching program is subdivided into 12 different constitutive modules. Each module is performed for one week and contains a particular exercise, which is supported by a corresponding daily SMS reminder. The reminder contains adapted information to obtain the motivation and to impart daily tips. The coaching program also offers a variety of printing material (emergency plan, relaxation exercises, questionnaires, information, self-agreements etc.) which is connected to the respective exercise and includes interactive buttons, video clips and learning progress quizzes to examine the learning success. At the end of each week participants are asked to give feedback via the internet concerning their condition and level of motivation and whether or not they did their weekly task. Participants can also communicate among themselves through a forum or ask a HausMed team member in case they have any questions. The active monitoring (or rather supervising) of the entire twelve-week coaching course is carried out by the GP through a separate login account in a particularly secured physician area (motivation, condition and status of the module exercise). In addition to that, three specified telephone calls from the GP or a qualified practice nurse (week 2, 4 and 12) are implemented to primarily motivate and support the participants. If either a participant’s motivation or condition declines notably within the weekly feedback at any point during the coaching period or the module exercise is not completed, additional counselling from the GP or practice nurse is made over the telephone."
5-ix) Describe use parameters
"There is no limitation to the frequency of website use but participants were given a goal of using the website at least once a week and GPs are advised to log in into the program twice a week."
5-x) Clarify the level of human involvement
"Participants can also communicate among themselves through a forum or ask a HausMed team member in case they have any questions. The active monitoring (or rather supervising) of the entire twelve-week coaching course is carried out by the GP through a separate login account in a particularly secured physician area (motivation, condition and status of the module exercise). In addition to that, three specified telephone calls from the GP or a qualified practice nurse (week 2, 4 and 12) are implemented to primarily motivate and support the participants. If either a participant’s motivation or condition declines notably within the weekly feedback at any point during the coaching period or the module exercise is not completed, additional counselling from the GP or practice nurse is made over the telephone."
5-xi) Report any prompts/reminders used
"...daily SMS reminders, self-monitoring via internet and, finally, through an active monitoring and approximately 3 telephone calls during the 12 weeks by the GPs or their respective staff."
5-xii) Describe any co-interventions (incl. training/support)
"an active monitoring and approximately 3 telephone calls during the 12 weeks by the GPs or their respective staff."

6a) CONSORT: Completely defined pre-specified primary and secondary outcome measures, including how and when they were assessed
"The primary outcome measure was biochemically confirmed smoking status twelve weeks after inclusion into the study by use of a cotinine urine test. Secondary outcome measures were self-reported smoking status, weight, number of smoked cigarettes per day, physical activity and breathing difficulties...After decision of the GP that the participation of a patient was recommendable, an information form was given and discussed with the patients and a participation form had to be signed. At the same time the baseline data acquisition took place. All participants were asked to fill in a standardized questionnaire together with the GP... Participants of both groups were requested to document after twelve weeks the follow-up evaluation together with their physician. "

6a-i) Online questionnaires: describe if they were validated for online use and apply CHERRIES items to describe how the questionnaires were designed/deployed
We did not use online questionnaires.

6a-ii) Describe whether and how "use" (including intensity of use/dosage) was defined/measured/monitored
We did not monitored the "programme use" due to the privacy protection.

6a-iii) Describe whether, how, and when qualitative feedback from participants was obtained
We did not document feedbacks.

6b) CONSORT: Any changes to trial outcomes after the trial commenced, with reasons
"No changes were made on the trial outcomes within the entire study period."

7a) CONSORT: How sample size was determined

7a-i) Describe whether and how expected attrition was taken into account when calculating the sample size
"Sample size calculation was performed with G * Power 3 correcting for the cluster design (intra-cluster correlation coefficient = 0.05, average cluster size = 3) for two-sided testing (α = 5% and a power of 80%). For the expected effect were abstinence rates of 30% versus 10% assumed. Using these assumptions the calculated total sample size for primary outcome smoking cessation was 152 participants. Taking expected attrition into account we aimed at recruiting a total of 180 participants in about 80 general practices."

7b) CONSORT: When applicable, explanation of any interim analyses and stopping guidelines
We did not conduct interim analyses.

8a) CONSORT: Method used to generate the random allocation sequence
"All interested GPs were sequentially registered for randomization. After giving written consent, the participating practices were randomized either to the interventional or the control arm. The sequence of randomization (allocation 1:1) was provided by a methodologist, who did not participate in the execution of the study, via the program Randomizer (www.randomizer.org). Randomization was concealed by using sequentially numbered, opaque sealed envelopes hold by the study coordinator. Before starting recruitment of patients, physicians and practice nurses received detailed instructions by the research team on the study process (both intervention and control group) and on the coaching program (only intervention group). "

8b) CONSORT: Type of randomisation; details of any restriction (such as blocking and block size)
"a cluster-randomized controlled trial... the participating practices were randomized either to the interventional or the control arm..."
The randomisation tooks place on practice level and not on patients level.

9) CONSORT: Mechanism used to implement the random allocation sequence (such as sequentially numbered containers), describing any steps taken to conceal the sequence until interventions were assigned
"The sequence of randomization (allocation 1:1) was provided by a methodologist, who did not participate in the execution of the study, via the program Randomizer (www.randomizer.org). Randomization was concealed by using sequentially numbered, opaque sealed envelopes hold by the study coordinator. "

10) CONSORT: Who generated the random allocation sequence, who enrolled participants, and who assigned participants to interventions
"The sequence of randomization (allocation 1:1) was provided by a methodologist, who did not participate in the execution of the study, via the program Randomizer (www.randomizer.org). Randomization was concealed by using sequentially numbered, opaque sealed envelopes hold by the study coordinator...The GPs were requested to recruit individuals with the desire for smoking cessation and for whom smoking cessation was recommendable. "

11a) CONSORT: Blinding - If done, who was blinded after assignment to interventions (for example, participants, care providers, those assessing outcomes) and how

11a-i) Specify who was blinded, and who wasn't
"The study was designed as a two-armed unblinded cluster-randomized controlled trial"

11a-ii) Discuss e.g., whether participants knew which intervention was the "intervention of interest" and which one was the "comparator"
"... the randomization of the present study was conducted on practice level before individual participants were included. Thus, physicians knew whether they recruited patients for the intervention or the control group which could lead to bias."

11b) CONSORT: If relevant, description of the similarity of interventions
"Individuals recruited by general practitioners randomized to the intervention group participated in a web-based coaching program ... Participants in the control group received usual care and advice from their practitioner without the web-based coaching program."

12a) CONSORT: Statistical methods used to compare groups for primary and secondary outcomes
"Group differences were calculated for all participants whose smoking status was available at baseline and follow-up (completer collective). Sensitivity analysis was performed by an intent-to-treat analysis assuming that participants with missing values had no smoking status change at all. The strongly variable cluster size caused major numerical problems in the linear mixed model analysis. As it was not possible to adjust for intra cluster correlations properly and because of the high variability of patients in practices, it was decided to perform the main analysis using Fisher's exact test without accounting for the clusters. For the main outcome smoking status we also performed secondary analyses based on logistic regression analyses with adjustment for age, gender, height and number of cigarettes smoked per day. We further conducted generalized estimating equations as sensitivity analysis to take account of practices as patient clusters."

12a-i) Imputation techniques to deal with attrition / missing values
"Sensitivity analysis was performed by an intent-to-treat analysis assuming that participants with missing values had no smoking status change at all."

12b) CONSORT: Methods for additional analyses, such as subgroup analyses and adjusted analyses
"The strongly variable cluster size caused major numerical problems in the linear mixed model analysis. As it was not possible to adjust for intra cluster correlations properly and because of the high variability of patients in practices, it was decided to perform the main analysis using Fisher's exact test without accounting for the clusters. For the main outcome smoking status we also performed secondary analyses based on logistic regression analyses with adjustment for age, gender, height and number of cigarettes smoked per day. We further conducted generalized estimating equations as sensitivity analysis to take account of practices as patient clusters."

RESULTS

13a) CONSORT: For each group, the numbers of participants who were randomly assigned, received intended treatment, and were analysed for the primary outcome

"Originally 92 practices were interested to participate and were randomised. 16 practices withdrew early after randomization (7 GPs from the intervention and 9 GPs from the control group), 34 practices (19 GPs from the intervention and 15 GPs from the control group) did not recruit any participant for the study (Figure 1). Altogether, 168 patients were recruited in 42 practices (86 patients in 20 intervention practices; 82 patients in 22 control practices). "

13b) CONSORT: For each group, losses and exclusions after randomisation, together with reasons

"..16 practices withdrew early after randomization (7 GPs from the intervention and 9 GPs from the control group), 34 practices (19 GPs from the intervention and 15 GPs from the control group) did not recruit any participant for the study...35 participants in the intervention group did not show up for the measurement at 12 weeks. In the usual care group 12 participants had missing values at 12 weeks, 1 of them had a missing baseline value. " We did not document any reasons for drop-out.

13b-i) Attrition diagram

"Figure 1"

14a) CONSORT: Dates defining the periods of recruitment and follow-up

The exact data are known and will be submitted later.

14a-i) Indicate if critical “secular events” fell into the study period

There are no “secular events” known.

14b) CONSORT: Why the trial ended or was stopped (early)

The planned time was over.

15) CONSORT: A table showing baseline demographic and clinical characteristics for each group

Table 2

15-i) Report demographics associated with digital divide issues

Table 2

16a) CONSORT: For each group, number of participants (denominator) included in each analysis and whether the analysis was by original assigned groups

16-i) Report multiple “denominators” and provide definitions

"Altogether, 168 patients were recruited...For 121 participants (51 from the intervention and 70 from the control group) information on smoking status was available both at baseline and after 12 weeks (complete-case)" and Table 3.

16-ii) Primary analysis should be intent-to-treat

"Group differences were calculated for all participants whose smoking status was available at baseline and follow-up (completer collective). Sensitivity analysis was performed by an intent-to-treat analysis assuming that participants with missing values had no smoking status change at all."

17a) CONSORT: For each primary and secondary outcome, results for each group, and the estimated effect size and its precision (such as 95% confidence interval)

"The self-reported cessation rate among the intervention group participants was 17.6 % (n=9/51) and among the control group participants 14.3 % (n=10/70) without a significant group difference (P=0.623). A logistic regression without adjustment (OR=0.78; 95% CI: 0.29; 2.08) and after adjustment for age, gender and number of cigarettes smoked per day at baseline (OR=0.62; 95% CI: 0.22; 1.78) revealed similar results. Within the intent-to-treat analysis self-reported cessation rate among the intervention group was 10.5 % (n=9/86). The self-reported cessation rate of the control group was 11.3 % (n=10/82). Results from the logistic regression without adjustment (OR=1.19; 95% CI: 0.46; 3.09) and after adjustment for age, gender, height and number of cigarettes smoked per day at baseline (OR=1.04; 95% CI: 0.39; 2.80) were similar...The cessation rate by use of the biochemical validation was for the intervention group 9.8% (n=5/51) and for the control group from 8.5% (n=6/70). Results were similar from the logistic regression without adjustment (OR=0.86 95% CI: 0.25; 3.0) and after adjustment for age, gender and number of cigarettes smoked per day at baseline (OR=0.63; 95% CI: 0.17; 2.40). The secondary analysis using generalized estimating equation showed also a non-significant result (P=0.74). Within the intent-to-treat analysis confirmed cessation rate among the intervention group was 5.8 % (n=5/86) and for the control group 7.3% (n= 6/82). Results from the logistic regression without adjustment (OR=1.28; 95% CI: 0.38; 4.36) and after adjustment for age, gender and number of cigarettes smoked per day at baseline (OR=1.01; 95% CI: 0.28; 3.62) were similar (Table 3)."

17a-i) Presentation of process outcomes such as metrics of use and intensity of use

We did not document process outcomes.

17b) CONSORT: For binary outcomes, presentation of both absolute and relative effect sizes is recommended

"The cessation rate by use of the biochemical validation was for the intervention group 9.8% (n=5/51) and for the control group from 8.5% (n=6/70). Results were similar from the logistic regression without adjustment (OR=0.86 95% CI: 0.25; 3.0) and after adjustment for age, gender and number of cigarettes smoked per day at baseline (OR=0.63; 95% CI: 0.17; 2.40). The secondary analysis using generalized estimating equation showed also a non-significant result (P=0.74). Within the intent-to-treat analysis confirmed cessation rate among the intervention group was 5.8 % (n=5/86) and for the control group 7.3% (n= 6/82). Results from the logistic regression without adjustment (OR=1.28; 95% CI: 0.38; 4.36) and after adjustment for age, gender and number of cigarettes smoked per day at baseline (OR=1.01; 95% CI: 0.28; 3.62) were similar (Table 3)."

18) CONSORT: Results of any other analyses performed, including subgroup analyses and adjusted analyses, distinguishing pre-specified from exploratory

"The result from the secondary outcome number of cigarettes smoked per day revealed within in the complete-case analysis that the mean difference after 12 weeks of the intervention group were about 2.7 cigarettes lesser than the mean difference of the control group (95% CI: -5.33; -0.58; P=0.045). After adjustment for number of cigarettes smoked per day at baseline, age, gender and height the difference between groups was no longer significant (95% CI: 0.27; 4.72; P=0,080). There were no statistically significant group differences for other secondary outcomes like weight, physical activity, use of nicotine replacement therapy and breathing difficulties (Table 4). "

18-i) Subgroup analysis of comparing only users

"Group differences were calculated for all participants whose smoking status was available at baseline and follow-up (completer collective). Sensitivity analysis was performed by an intent-to-treat analysis assuming that participants with missing values had no smoking status change at all."

19) CONSORT: All important harms or unintended effects in each group

These data are known and will be submitted later.

19-i) Include privacy breaches, technical problems

These data are known and will be submitted later.

19-ii) Include qualitative feedback from participants or observations from staff/researchers

These data were not documented.

DISCUSSION

20) CONSORT: Trial limitations, addressing sources of potential bias, imprecision, multiplicity of analyses

20-i) Typical limitations in ehealth trials

"However, some important methodological aspects for the interpretation of the study results need to be considered. First of all, the randomization of the present study was conducted on practice level before individual participants were included. Thus, physicians knew whether they recruited patients for the intervention or the control group which could lead to bias. Secondly, due to the highly variable cluster sizes the statistical analysis of our data was not straightforward. Classical linear mixed models taking the cluster design into account could not be used because of numerical problems. Therefore we used simple Fishers' exact test (which ignores intra-cluster correlation) and an additional multilevel analysis (which runs with problems when cluster sizes differ) as sensitivity analysis. Thirdly, according to our power calculations the target number of participants was not completely reached which may have reduced the study's ability to detect significant differences between the groups. Fourthly, the proportion of participants without follow-up values was undoubtedly higher in the intervention than in the usual care group. This could be partly due to the fact that participants in the control group received a small financial incentive while those in the intervention group did not. Participants in the intervention group might also have been less willing to have an additional practice visit after completing the program than those in the control group who had little practice contact otherwise. Therefore, our complete case analysis with the self-reported cessation might overestimate the rates to some extent. Within the intent-to-treat analysis, where the missing post values were replaced without a change of smoking status (baseline carried forward), the cessation rates were clearly smaller. Fifthly, the content of the usual care was not further evaluated. The practitioners of the control group were only asked to change nothing in their usual way of counselling and to treat their participants in the same manner as usual. There was no additional documentation of their counselling provided. "

21) CONSORT: Generalisability (external validity, applicability) of the trial findings

21-i) Generalizability to other populations

"Strengths of the present study were the embedding of the study in a realistic primary care setting and the use of a biochemical validation of the self-reported cessation status."

21-ii) Discuss if there were elements in the RCT that would be different in a routine application setting

"the embedding of the study in a realistic primary care setting" imply that the difference to the routine application will be very small.

22) CONSORT: Interpretation consistent with results, balancing benefits and harms, and considering other relevant evidence

22-i) Restate study questions and summarize the answers suggested by the data, starting with primary outcomes and process outcomes (use)

"We found that the web-based coaching program in combination with an accompanied telephone counselling and monitoring in general practice was not effective for achieving smoking cessation compared to usual care. There were no significant differences found between the groups. However, the present quit rates are clearly higher compared to the spontaneous quit rates which are stated by the majority with about 1 %"

22-ii) Highlight unanswered new questions, suggest future research

"Further RCTs are desirable in order to investigate in larger populations and in long-term outcomes as well as in the contents of usual primary care."

Other information

23) CONSORT: Registration number and name of trial registry

German Register for Clinical Trials, registration number DRKS00003067

24) CONSORT: Where the full trial protocol can be accessed, if available

The trial protocoll can be request from the funding company "HausMed eHealth Services GmbH"

25) CONSORT: Sources of funding and other support (such as supply of drugs), role of funders

"This study was completely funded by HausMed eHealth Services GmbH (Berlin, Germany). "

X26-i) Comment on ethics committee approval

"The study was approved by the Medical Ethics Committee of the Technische Universität München (on 19 April 2011) and was in accordance with ethical standards for human experimentation established by the Declaration of Helsinki."

x26-ii) Outline informed consent procedures

"All participants gave written informed consent... an information form was given and discussed with the patients and a participation form had to be signed. "

X26-iii) Safety and security procedures

"A data and safety monitoring board was established before the beginning of the study...Participants can also communicate among themselves through a forum or ask a HausMed team member in case they have any questions...The active monitoring (or rather supervising) of the entire twelve-week coaching course is carried out by the GP "

X27-i) State the relation of the study team towards the system being evaluated

"The sponsor did not have access to study data and did not influence the development of this manuscript. AS, KL, MM, SW are employed at University Hospital Klinikum rechts der Isar, Technische Universität München. MH is medical student. There were no other financial and non-financial competing interests. "
